# Wear Risk Prevention and Reduction in Total Hip Arthroplasty. A Personalized Study Comparing Cement and Cementless Fixation Techniques Employing Finite Element Analysis

**DOI:** 10.3390/jpm11080780

**Published:** 2021-08-10

**Authors:** Carlos González-Bravo, Miguel A. Ortega, Julia Buján, Basilio de la Torre, Loreto Barrios

**Affiliations:** 1 Department of Medicine and Medical Specialities, Faculty of Medicine and Health Sciences, Ramón y Cajal Institute of Sanitary Research (IRYCIS), University of Alcalá, Alcalá de Henares, 28034 Madrid, Spain; cgbravo@amasi.es (C.G.-B.); miguel.angel.ortega92@gmail.com (M.A.O.); mjulia.bujan@uah.es (J.B.); loreto@lycea.es (L.B.); 2 A+I Architecture and Engineering Ltd., 28224 Madrid, Spain; 3 Department of Surgery, Medical and Social Sciences, Faculty of Medicine and Health Sciences, Ramón y Cajal Institute of Sanitary Research (IRYCIS), University of Alcala, Alcala de Henares, 28034 Madrid, Spain; 4 Department of Orthopedic Surgery, University Hospital Ramón y Cajal, 28034 Madrid, Spain

**Keywords:** total hip arthroplasty, finite element method, cemented and uncemented acetabular fixation, polyethylene wear patterns, cervical–diaphyseal angle, center of rotation, material head, size head, liner thickness

## Abstract

The wear rate on Total Hip Arthroplasty (THA) entails a heavy burden for patients. This becomes more relevant with increased wear risk and its consequences such as osteolysis. In addition, osteolysis has been described in cemented and uncemented acetabular implants, and nowadays, controversy remains as to whether or not to cement the acetabular component. A personalized theoretical study was carried out to investigate which parameters have an influence on wear risk and to determine the best fixation method. Liner wear risk was assessed for two different types of fixation (cemented vs uncemented) through Finite Elements Analysis (FEA). The intraoperative variables used to determine the wear risk (cervical-diaphyseal angle, Center of Rotation positioning -COR-, head material, head size, and liner thickness) are vital parameters in surgical planning. Two types of tridimensional liner models of Ultra High Molecular Weight Polyethene (UHMWPE) were simulated through finite element analysis (FEA—over 216 cases were the core of this research). A significant relationship was found between the cervical-diaphyseal angle and wear risk (*p* < 0.0001), especially in valgus morphology. The acetabular fixation technique (*p* < 0.0001) and liner thickness (*p* < 0.0001) showed a significant relationship with wear risk. According to our study, using a cemented fixation with a thick liner in the right center of rotation appears to be the proper stratagy for preventing polyethylene liner wear.

## 1. Introduction

THA is an accepted and successful procedure for patients suffering from degenerative hip joint disease. Once the entire joint is replaced with an artificial one, a new variable is introduced in patients’ regular activity regardless of age: wear on the polyethylene liners. At present, there seems to be a debate regarding the ideal method of fixation for the liner [1,2]. However, some authors [3] claim that the cemented fixation is the “gold standard” with multiple papers showing a relationship between uncemented cases and an increased wear rate [4,5,6,7,8].

Polyethylene wear is influenced by different parameters such as the center of rotation (COR) location, the femoral head size and material, or the liner thickness. These parameters affect clinical outcomes following hip arthroplasty [4,9].

Cervical–diaphyseal angle (varus or valgus morphology) plays a critical role in the stresses generated at the bearing surfaces [10]. However, studies regarding the role of the cervical–diaphyseal angle on liner wear are absent in the literature.

Nowadays, there is a lack of intensive computational studies and no quantified data on wear risk regarding the aforementioned parameters in cemented or uncemented acetabular fixation. Many studies are limited by the heterogeneity of patients and treatments. This lack of uniformity in clinical studies makes it difficult for surgeons to draw conclusions relevant to their clinical practice.

A personalized study can assess this critical parameter related to specific morphology of the hip joint. The wear risk prevention on the artificial hip joint for these patients could begin with a set of numerical simulations implementing general and particular parameters.

Bearing this in mind, the present research has developed a numerical wear simulation using FEA to check distinct features of wear risk [11,12,13,14,15,16,17,18] with particular focus on the variables that affect the integrity of the liners. One of the most powerful tools in the computational scenario is obtaining an order of magnitude to determine and prevent the causes of wear rate for singular patients and, consequently, to avoid osteolysis progress and failure in THA.

Good decision-making for orthopedic surgeons in a distinctive THA plan is the best wear reduction strategy. The purpose of this study is to quantify the role of previously described parameters in polyethylene wear through a numerical method (FEA).

## 2. Materials and Methods

**Figure 1:**
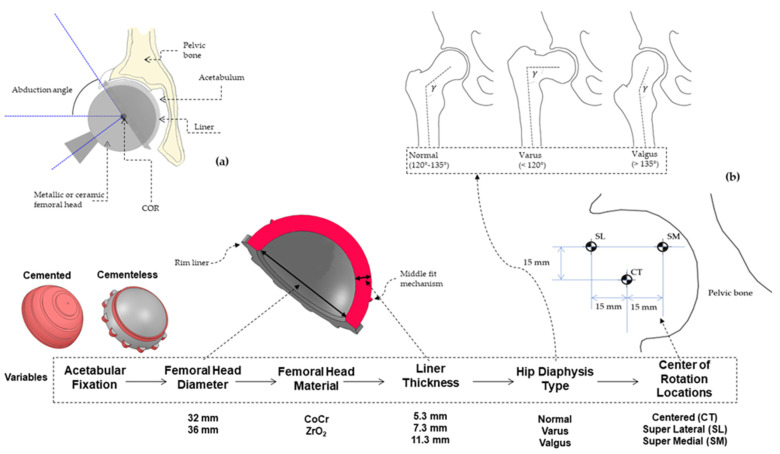
(**a**) Common parts of a Total Hip Arthroplasty (lateral cross-section). (**b**) Summary of parameters and variables analyzed with Cervical-Diaphyseal Angle in Normal, Varus and, Valgus.

For the present study, a set of simulations (216) were carried out over the 3D liners modeled in version 2017 of SOLIDWORKS^®^ (Dassault Systèmes, Vélizy-Villacoubla, France) from real geometry of the Neutral (E1 & ArComXL) G7 acetabular system (in the case of cementless fixation) and Exceed ABT (in the case of cemented fixation) currently marketed by Zimmer Biomet in a Ultra High Molecular Weight Polyethylene (UHMWPE) material. The contact between liner and cup or bone was not assessed in this study and neither was the liner and femoral head contact. However, the last issue was taken into account through the Hertz theory, as shown below. Since the number of possibilities was large, two standard head femoral diameters (32 mm and 36 mm) generally used by surgeons were chosen and, within these, three different thicknesses: one close to the minimum (5.3 mm), the second in the middle range (7.3 mm) and, the last close to maximum (11.3 mm). For a general sketch of variables and values, see Figure 1a and Figure 1b. Liner material (Table 1 (a)) was considered isotropic as far as its mechanical behavior was concerned. Furthermore, no large displacement was set up in order to obtain the elastic range of results, avoiding nonlinearities in the FEA. With this in mind, a general comparison is possible when it comes to determining the elastic limit (around 25 MPa) of the UHMWPE and, therefore, enabling study of the wear risk in those particular areas of the liner when the von Mises (VM) stress is analyzed, as we will see in the results section.

**Figure 2:**
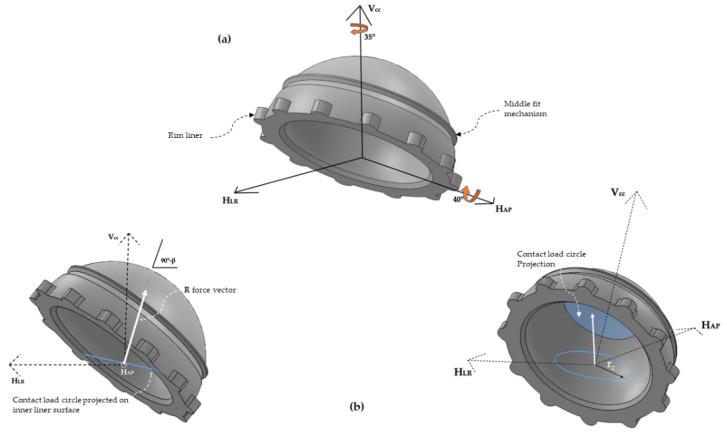
(**a**) 3D liner modeled with its orientation in the three-axis: HLR (Horizontal Left-Right or frontal); HAP (Horizontal Anteroposterior, Sagittal o Dorsoventral); VCC (Vertical Craniocaudal). (**b**) 3D liner with load vector R applied over the contact Hertz theory circle.

To simulate a real orientation of the liner inside the acetabulum, 3D models were aligned (Figure 2a) with particular directions of an abduction angle around the H_AP_ axis (40°) and an anatomical orientation in acetabular direction (35°) around the V_CC_.

The cup angle (40°) was fixed according to the literature [17,19,20,21,22,23,24,25] considering that a cup inclination angle greater than 45 degrees is associated with increased wear rates.

As far as Femoral Head Material is concerned, a Contact Hertz Theory [26,27] was used to determine areas of forces contact in both metallic (CoCr) and Ceramic (ZrO_2_) which have been taken into account in this study by their mechanical properties (Table 2). These properties include friction coefficients for the metal-UHMWPE and ceramic-UHMWPE for each femoral head size taken from previous studies [26,28] and are needed to determine the shear force applied on the spherical surface of the inner liner by means of the contact circle areas (Figure 2b). The contact circle area calculated through Hertz contact theory was projected in the R vector force direction as explained below. Thus, a contact area is created over the inner side of the liner (curve geometry) from a plane circle in the right direction and area location of the force application (R) and with the correct size determined thanks to Hertz theory, as explained below.

To calculate the circle area, it was necessary to apply Equations (1) to Equation (3) where r_c_ (Figure 2b) is the contact radio circle projected in the R force direction that includes the properties of femoral head materials.

(1) $$r_{c}=\sqrt[{3}]{\frac{Rr_{e}}{4E_{e}}}$$

(2) $${r_{e}}^{-1}=\frac{1}{r_{\mathrm{fh}}}-\frac{1}{r_{\ln}}$$

(3) $${E_{e}}^{-1}=\frac{1-\nu_{\mathrm{fh}}}{E_{\mathrm{fh}}}-\frac{1-\nu_{\ln}}{E_{\ln}}$$

where R is the total force over the hip, re is the equivalent radius equation and E_e_ is the equivalent elasticity modulus obtained from Equations (2) and (3), respectively. Therefore, E_fh_ and E_ln_ belong to the femoral head and liner elasticity modulus for both materials and, in turn, r_fh_ and r_ln_ correspond to the femoral head and liner radii. Finally, ν_fh_ and ν_ln_ stand for fricction coefficient of the femoral head and liner respectively. The minus sign between fractions is due to the kind of convex–concave (femoral head-liner) contact.

### 2.1. Load and Boundary Conditions

A different total force vector (R) over the liner geometries was considered for each combination of COR positioning (Center, Super Lateral, and Super Medial) and cervical-diaphyseal angle (varus, valgus or normal).

**Figure 3:**
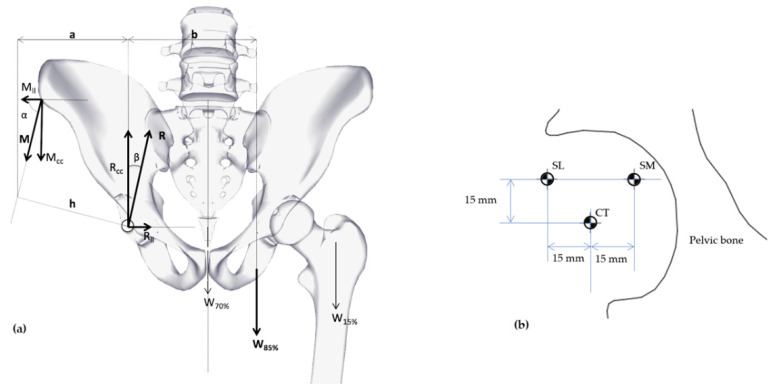
(**a**) Biomechanics diagram forces over hip (frontal projection). (**b**) Displacement for CT, SL, SM as COR positioning.

The body weight (W) was assumed constant with 800 N and used with reduction (W85%), following other authors [29,30]. Values a and b (Figure 3a) were taken from a different set that corresponds with Valgus, Varus, and Normal (Hip Diaphysis Type) and COR positioning (Figure 3b) as Super Lateral (L) Center (CT) and Super Medial (SM).

(4) h=asinα

(5) $$M=\frac{W_{85\%}b}{h}$$

(6) $$R=\frac{M\sin\alpha +W_{85\%}}{\cos\beta }$$

(7) $$\tan\beta =\frac{M\cos\alpha }{M\sin\alpha +W_{85\%}}$$

The Equations (4)–(7) depict the balance of the free body (Figure 3a) with which it is possible to assembly all data (Table 1 (c)). Figure 3b shows the COR location where Superior Lateral (SL) and Medial (SM) locations have both constant vertical value (15 mm) and horizontal constant value (15 mm) from the Center (CT) positioning.

As is shown in Table 1 (c), the gluteus medius vector angle (α) is fixed in each group of values for the three cervical–diaphyseal angles and stems from the geometrical structure of the varus hip (19º below the average value) and valgus hip (7° above the standard value) when the angle is varied [31,32] from 71°.a = 68 mm, given from Le Veau [33].

As aforementioned, specific vector loads were applied over the inner side of the liners depending on their cervical–diaphyseal angle and COR positioning (Table 1 (c)). These loads, which depict the femoral head sphere, were distributed (Figure 2b) all over the circle area, and the dimensions were previously calculated from Hertz contact theory. This area is the contact surface between the ball femoral head and the inner side of the liner and implies a significant reduction of time computed to obtain a desirable order of magnitude in the outcomes.

**Figure 4:**
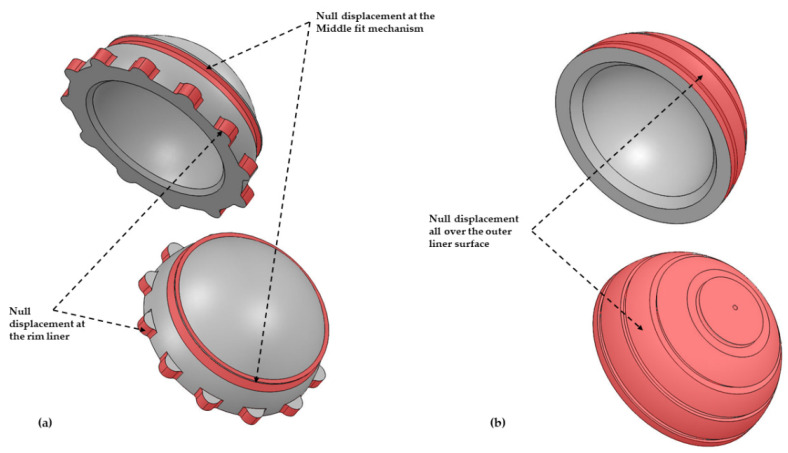
3D model liner with boundary conditions. (**a**) Cementless acetabular fixation. (**b**) Cemented acetabular.

Finally, a cemented acetabular fixation was configurated over the models (ABT geometry) through a complete restriction (Figure 4b) of movements (no displacements on or turns around three spatial axes) of the outer surface including the rim of the liner. The other condition (uncemented) was succeeded by partial rim restriction and the middle fit mechanism on the shell (Figure 4a) with G7 geometry. This kind of restriction avoids nonlinearity created by possible friction contacts if we consider a shell material. To sum up, this research considers the shell and bone mechanical properties, applying these specific boundary conditions over the liner geometry (G7 and ABT). The inner geometry is the same in both cases, but the outer geometry is different.

### 2.2. FE Modeling and Simulations

Structural static simulations by FEA were carried out over the isotropic behavior of the liner material. This kind of election for the general study is in order to develop a wide range of von Mises results, especially considering the order of magnitude such a numerical tool can give. The simulation software was an iterative solver from SOLIDWORKS 2017. However, results were compared to other software (ANSYS Workbench 19 R2) with equal conditions and parameters (size and kind of elements) with a negligible difference (1.02% using tetrahedron elements and 0.2% using hexahedron elements) as far as the VM stress result is concerned.

Since the number of simulations (216) was extremely high and the results were in a similar order of magnitude between both solvers (SOLIDWORKS and ANSYS Workbench), all simulations were carried out on SOLIDWORKS iterative solver. Other researchers [34] have used the same software as in this research to analyze wear risks in the liner with similar results.

Despite that the size element was the same in all simulations (Table 2), the number of elements (and nodes) was increased from around 55,010 elements in the most miniature liner to 159,946 elements in the biggest liner. On the other hand, the element aspect ratio of values less than 3 was, in all simulations, above 99%.

The election of the solid tetrahedron as a meshing element with an automatic transition to curved shapes over the spherical geometry of liners provides accurate identification of maximum VM points, especially in cementless fixation, since the maximum value was not always in the inner surface of the contact load, as we will discuss in the results.

Finally, to arrive at accurate results, eight Jacobian points were selected in all simulations. The iterative convergence solver spent around 10.4 h of computer time in all simulations using a microprocessor with four cores.

### 2.3. Wear Risk and Statistical Analyses

VM stress, stress intensity factor (SI) and other results for prediction of wear risk is often used by authors [18,35] as an order of magnitude. VM stress is more reliable as a predictor of wear rate since this kind of criterion comprises the three principal direction stresses in one equation with a long track of approximation to the actual behavior of materials with ductile crack. Therefore, in this research, VM stresses are assumed as a wear risk tester and then contrasted to experimental data from different authors to validate values and locations of maximum points for that kind of stress.

Multiple regression was carried out for statistical analyses running all variables under SPSS software, version 13.0 (SPSS Inc., Chicago, IL, USA). VM Stress was fixed as a dependent variable, and ANOVA analyses were used to determine the *p*-value for all variables. Due to the literature for some variables being quite limited as far as the statistical population is concerned, 1% was considered of statistical significance. However, as mentioned earlier, an order of magnitude was considered to approximate the real significance of all variables.

## 3. Results

### 3.1. VM Stress vs. SI Stress

**Figure 5:**
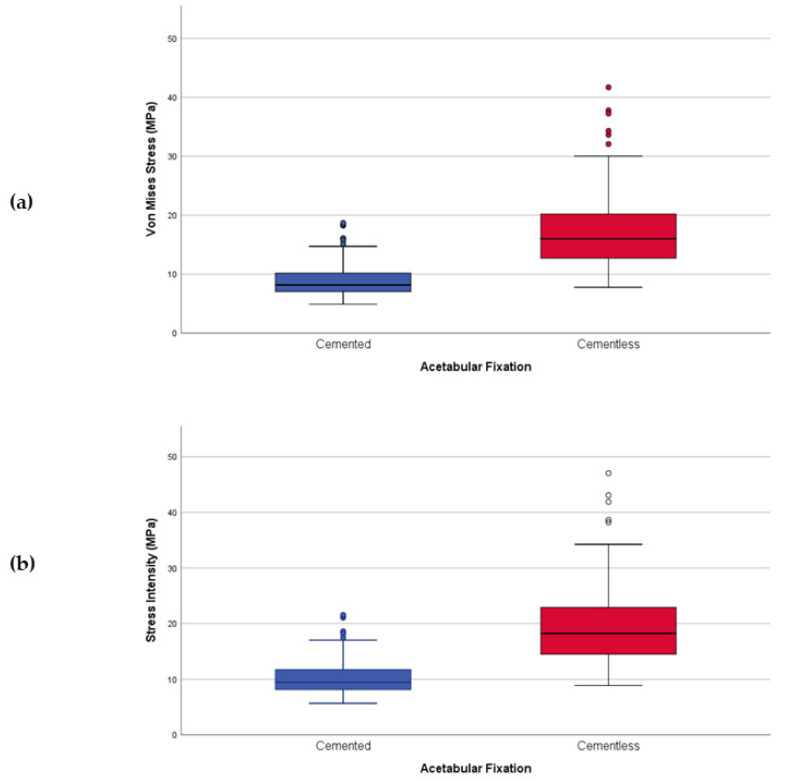
Acetabular Fixation wear risk based on stresses; (**a**) VM Stress; (**b**) SI stress.

In general, results reveal no difference between VM and SI stresses (*r*^2^ = 1) when we analyze outcomes as a whole. In a more particular view and taking into account the acetabular fixation, we can observe that the correlation between both types of stresses (WM and SI) depicts a slight difference. VM stress (Figure 5a) is a little lower (8.19%) than SI stress (Figure 5b) even in terms of wear risk probability. The equivalent is tangible when we analyze the acetabular fixation’s general behavior in any parameter (cervical–diaphyseal angle, COR positioning, liner thickness, femoral head material, or femoral head size).

### 3.2. General Analyses of the Parameters

Considering a summary of statistical parameters, VM Stress as a variable dependent displays a *r*^2^ = 0.570 (Table 3 (a)) and its statistical significance is recorded through a *p*-value < 0.0001 (Table 3 (b)).

Results for valgus show a *p*-value < 0.0001 (Table 3 (c)) as the statistical significance between cervical–diaphyseal angle and wear risk.

The same is true for both acetabular fixation and thickness parameters with a *p*-value < 0.0001. Head Diameter showed a lesser significance (*p*-value = 0.001). The *p*-value for COR (*p*-value = 0.008) and head material (*p*-value = 0.012) showed a weaker relationship between those two parameters and wear risk.

On the other hand, a graphical comparative for all parameters shows the general behavior comparing cemented and uncemented acetabular fixation in which there is a lesser wear risk for cement fixation than for cementless. Besides, this tendency is common in all parameters since a low decrease is observed from values for cemented fixation, while, in comparison, the cementless has a more pronounced decrease of its sub-parameters.

**Figure 6:**
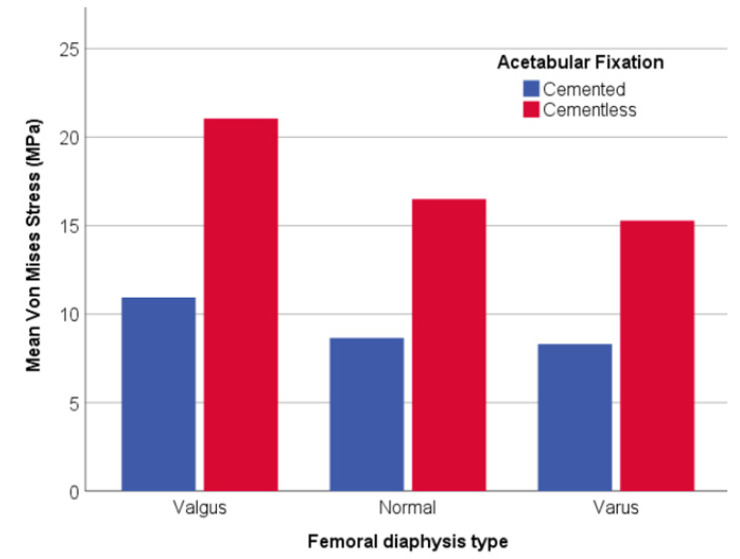
Bar graph with the relationship between VM Stress (MPa) and the femoral diaphysis type.

In the cervical–diaphyseal angle variable (Figure 6), valgus was more than 20 MPa for cementless fixation, which doubled the cemented model (around 10 MPa), being the highest values for each type in comparison with varus (15 MPa for cementless and 8 MPa for cemented).

**Figure 7:**
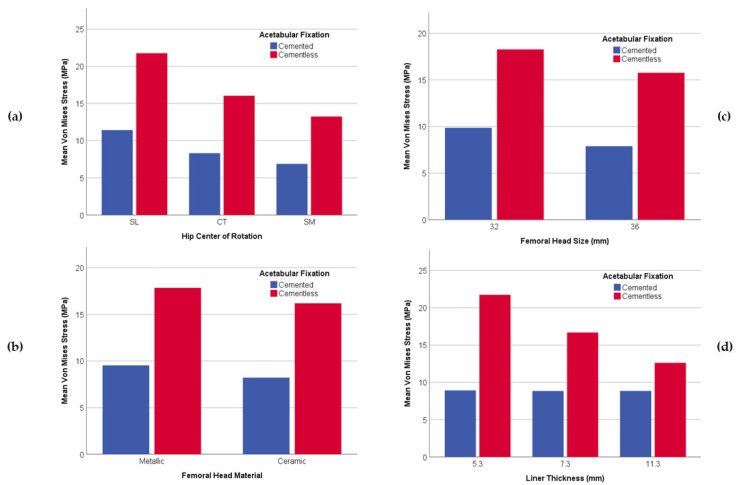
Bar graph with the relationship between variables and VM Stress (MPa); (**a**) VM Stress and Hip Center of Rotation; (**b**) VM Stress and Femoral Head Material; (**c**) VM Stress and Femoral Head Size; (**d**) VM Stress and Liner Thickness.

As far as COR parameter (Figure 7a) is concerned, a mean VM Stress graph shows that Superior and Lateral (SL) location consistently exhibits the most significant value (over 18 MPa). An intermediate value is found for the Center (CT) location (12.50 MPa), and the lowest value is found on all occasions in Superior and Medial (SM) position (10.15 MPa).

Both femoral head material and size parameters (Figure 7b,c) manifest the same stress behavior with an insignificant difference between them. Nonetheless, there is a significant variation between cemented and uncemented fixation. The values for cemented fixation are much lower (below 10 MPa) than for cementless fixation (above 15 MPa).

Finally, VM Stress increased with a decrease in the liner thickness (Figure 7d) for uncemented fixation. However, VM stress remained nearly constant for the three different liner thicknesses for cemented fixation.

### 3.3. Stress Distribution over the Liner

**Figure 8:**
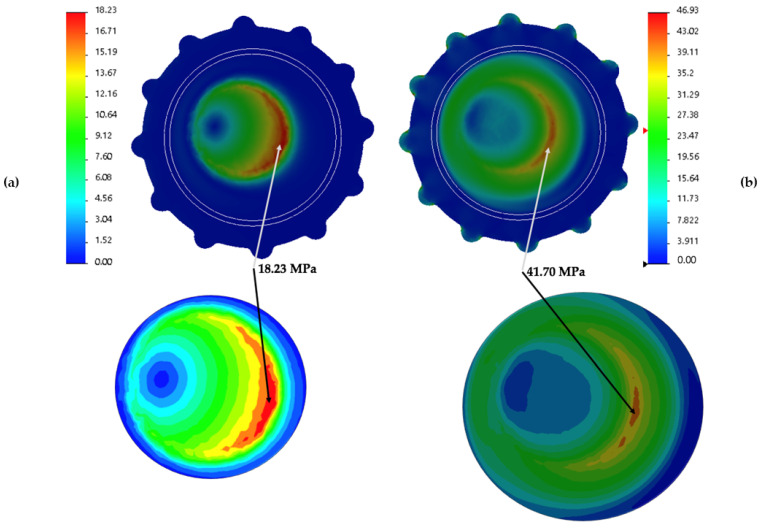
FEA results in images; (**a**) VM Stress cemented fixation of the inner liner surface; (**b**) VM Stress uncemented fixation of the inner liner surface.

The comparative analysis of stress distribution over the inner and outer surface of the liner (Figure 8) in cemented and cementless fixation depicts a particular stress map of each acetabular fixation with its stress range values in MPa. In order to apply the render in a specific case, a 32 mm head size was chosen made of CoCr with a thickness liner of 5.3 mm, located at Super Lateral COR position and with valgus cervical–diaphyseal morphology. Nonetheless, the general distribution of all liners analyzed follow the same stress map.

The inner surface of liners exhibits how the stress area changes from a more intensive location (Figure 8a) in cemented fixation to a more widespread distribution in cementless (Figure 8b). However, while the location of the stresses for cemented is entirely concentrated, its maximum value (the maximum of the whole liner) is shorter (18.23 MPa) than the uncemented fixation (41.70 MPa). In other words, on that side of the liner, the cement fixation is 43.72% of that of the cementless.

These values are in the same area in both types of fixation, but the cementless is not the area of maximum VM Stress value. A glance at the outer side of both sorts of fixation shows the same area of maximum VM Stress value for the cementless fixation.

**Figure 9:**
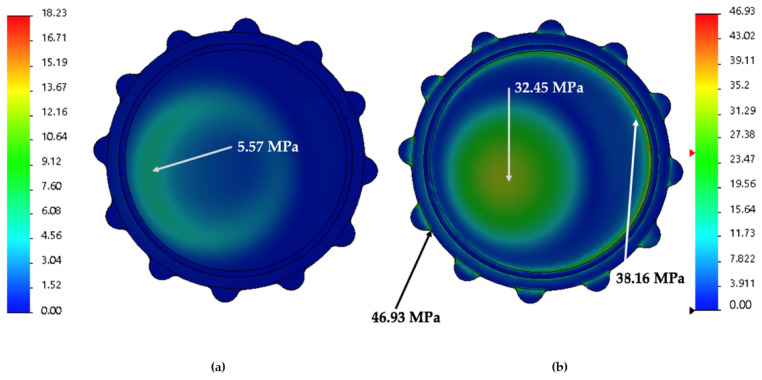
FEA results in images; (**a**) VM Stress cemented fixation of the outer liner surface; (**b**) VM Stress uncemented fixation of the outer liner surface.

The study of the outer surface (Figure 9) shows different VM Stress behavior. Cemented fixation (Figure 9a) develops a slight distribution in a half-moon shape with a maximum VM Stress in the middle of that shape (5.57 MPa), whereas the cementless (Figure 9b) has three different areas of VM stress.

The first stress area is located on the top of the liner (32.35 MPa) with a circle shape at the exact location where cemented fixation depicts a half-moon shape. The second area (38.16 MPa) matches a circle made in the liner and is responsible for its fitting assembly with the cup fixed on the pelvic bone. Finally, the third area (46.43 MPa) is located on the rim of the liner.

## 4. Discussion

Despite the general fact that THA is a well-accepted and reliable surgical procedure to return patients to proper function, aseptic loosening of implants, mainly of the acetabular component, due to polyethylene wear, continues to be a concern among orthopedic surgeons. A personalized theoretical study focused on cervical–diaphyseal morphology was run to obtain detailed results of these specific variables and of wear risk in patients who underwent a hip replacement.

This constitutes the first particularized study that quantifies the wear risk of polyethylene in Primary Hip Arthroplasty. Moreover, it guides the ideal reconstruction of the acetabular component, taking into account the different anatomical aspects of the patient. The results obtained are not intended to be an axiom in the surgical decision, but rather a reference for the hip surgeon regarding surgical planning.

Our findings roughly correlate with previous studies regarding the role of the type of fixation (cemented vs uncemented), COR positioning, femoral head size and material and liner thickness in wear risk. To the best of our knowledge, no previous studies have assessed the role of cervical–diaphyseal morphology in liner wear.

Several critical reviews have led to controversy regarding best acetabular fixation method. Nevertheless, three thorough reviews [1,2,3] suggested that a higher annual wear rate may be encountered in uncemented acetabular components when compared to cemented components. Moreover, according to Hartofilakidi et al., lytic lesions associated with uncemented acetabular components seemed to be more aggressive than those associated with cemented components [36]. This study confirms, from simulations and numerical data, that wear rate increases in UHMWPE liners with uncemented acetabular fixation.

The location of the osteolysis described for uncemented cups in previous studies [7,9,37] seems to have a similar distribution as the areas of stress described in our study (Figure 8 and Figure 9) for uncemented fixation. These areas are concentrated in the outer area of the liner, mostly in the liner-shell interface. This could be due to the high stress suffered by UHMWPE in contact with the metallic cup [13,38].

The significant difference between cemented and cementless fixations might be explained, from a contact mechanics perspective, due to both kinds of fixations’ specific boundary conditions. Stress distribution in each type of fixation is quite different, hence the strains along the liner thickness.

As regards the COR, several prior studies [18,39,40,41] have linked the elevation and lateralization positioning of the COR, with the failure of THA. For uncemented cups, Georgiades et al set the probability value for statistical significance at 5% [39]. They reported both parameters, lateral (*p* = 0.001) and superior (*p* = 0.049) positioning of the COR, as the responsible cause of wear rate and osteolysis around the acetabular component. Likewise, Hirakawa et al. drew a similar scenario in 2001 [19] with *p* < 0.0001 in lateral positioning and only *p* = 0.39 for superior positioning as statistical significance. Although we have analyzed superior positioning combined with lateral and medial displacement of COR, the statistical significance reached (*p* = 0.008) coinciding with previous clinical studies (Table 4). Our results suggest an increment (40%) of wear risk in SL positioning when compared to SM positioning (Figure 7a), while CT shows intermediate values.

This aspect is highly relevant in two particular clinical settings. It is essential to perform the acetabular reconstruction in the proper anatomical COR in patients with high hip dislocation sequelae, avoiding the superior placement. Likewise, in patients with hypertrophic arthritis of the hip, with a large medial osteophyte, it is necessary to ream in a medial direction to avoid the acetabular component’s superior and lateral placement.

With regard to the head material we find values close to 5% in the statistical significance, although prior studies such as Sato with *p* = 0.45 [42], Garvin with *p* = 0.58 [43], or Teeter with *p* = 0.32 [44] do not suggest a relationship between this variable and wear risk. Our results seem to suggest that there is less wear risk in ceramic head material than in metallic. However, Figure 7b provides a graphical analysis of the difference found in our study between CoCr an ZrO_2_, which hardly reached 8%.

Another ongoing debate in the literature is the relationship between the head size (diameter) and the wear rate. Again, Teeter declared an unequal distribution (between head size and wear risk) of femoral head size across all groups [42] studied in his research (*p* < 0.001 for ceramic CoCr and *p* = 0.055 for OxZr-CoCr). Other authors such as Bragdon with *p*-value in the range from *p* = 0.23 to *p* = 0.90 [45] and Lachiewicz with *p* = 0.593 [46] stated similar conclusions. Our research has studied size for 32 mm and 36 mm. We are under the impression that our results are easier to interpret with the help of bar graphs (Figure 7c) in which only a slight difference can be appreciated (15%–17%) between both diameters. Our findings do not support the general idea that a larger head size increases wear rate. These results have clinical relevance when a surgeon decides to use a large-diameter head to achieve a greater range of motion and stability. However, we must consider that this lack of difference found in our study regarding the head size could be explained due to the static structural analyses, in which there are no sliding distance considerations.

The correlation of liner thickness and wear rate has been the subject of many previous studies. Berry suggested that the use of thin liners along with uncemented cups and an acetabular abduction angle of more than 45° was a risk factor for polyethylene wear [4]. Astion [9] found an increase in stress contact (*p* = 0.03) related to decrease of liner thickness. Muratoglu [47], pointing in the same direction, recommended liners thicker than 5 mm. Shen reported an apparent contradiction between his data [35] from the FEA study (an increase of stress with a decrease of liner thickness) and his data from the hip simulator (apparently no significance stress-thickness with *p* = 0.17). Finally, Bartel suggested, after a FEA, that minimum plastic thickness of 4–6 mm should be maintained [38].

Surprisingly, our results point to no relationship between liner thickness and wear risk when it comes to cemented fixation. On the contrary, our results with cementless fixation resemble those previously cited. Considering this, using a cemented fixation could constitute a strategy to minimize the effect between both parameters (liner thickness and wear risk), as may be suggested by the findings showed in Figure 7d.

One of the main novelties of our study is the assessment of the role of the cervical–diaphyseal angle in wear risk. This parameter is usually treated as an inherent parameter for each patient in the literature. However, this parameter is influenced by surgeon decision making medializing or lateralizing the femur by using a standard or high-offset stem. Indeed, our results show that the higher the cervical–diaphyseal angle, the more wear risk. A 31% decrease in wear rate was found when comparing valgus hips (maximum values for cervical–diaphyseal angle) with varus hips (minimum values for cervical–diaphyseal angle). This finding may have relevance in clinical practice as patients with coxa valgus, prevalent in the sequela of hip dysplasia, may benefit from the use of “high offset” stems in order to reduce the wear risk of polyethylene.

Our study has several limitations that warrant consideration. Firstly, FEA modeling focused on wear prediction under a normal walking condition, but it did not evaluate other daily activities. Secondly, our study did not consider the dynamic aspect of the acetabular orientation since pelvic tilt, lumbo–pelvic kinematics and spine–hip relationship-adjusted cup alignment were not assessed. Lastly, wear FEA analysis of the liner simulated the dry contact between bearing surfaces, not taking into account the lubrication that exist under physiological conditions.

Despite these limitations, this study provides a quantification of the relationship between wear risk and five parameters closely correlated with polyethylene wear in previously conducted clinical studies. It also provides the first evidence that cervical–diaphyseal angle may affect polyethylene liner wear.

## 5. Conclusions

In conclusion, although this is a theoretical study, it constitutes a personalized approach to quantifying the effects of many variables on the wear polyethylene risk probability, especially concerning cervical–diaphyseal angle morphology and the two widespread currently acetabular fixations. It provides guidance for the orthopedic surgeon to plan the acetabular reconstruction in THA, preventing and reducing wear risk by the use of a cemented fixation with high polyethylene liner thickness, a femoral head equal to or greater than 32 mm, and a high-offset femoral stem.

## Figures and Tables

**Table 1:** (**a**) Mechanical properties of UHMWPE liner. (**b**) Mechanical properties of femoral head and equations of contact Hertz theory. (**c**) Biomechanics and geometrical values.

**(a)**													
**E ^1^ (MPa)**	**G ^2^ (MPa)**		**Ν ^3^**			**F_y_^4^ (MPa)**			**f_u_^5^ (MPa) 2**	**Strain Max (%)**			
940	322		0.46			25			40	500			
^1^ Modulus of Elasticity; ^2^ Modulus of Rigidity; ^3^ Ratio of Poisson; ^4^ Yield Strenght; ^5^ Ultimate Strenght.													
**(b)**													
**Material**	**E ^1^ (GPa)**				**ν ^2^**			**μ (32) ^3^**			**μ (36) ^4^**		
CoCr	210				0.30			0.133			0.14		
ZrO2	358				0.24			0.096			0.085		
^1^ Modulus of Elasticity; ^2^ Ratio of Poisson; ^3^ Fricction Coeffient for 32 mm of femoral head; ^4^ Fricction Coeffient for 36 mm of femoral head.													
**(c)**													
**Cervical-Diaphyseal Angle**		**COR ^1^**	**a ^2^ (mm)**	**b ^3^ (mm)**			**h ^4^ (mm)**	**α ^5^ (◦)**	**β ^6^ (◦)**			**M ^7^ (N)**	**R ^8^ (N)**
		SL ^9^	53	125			45.23	71	13.98			1879.33	2531.98
**Normal**		CT ^10^	68	110			64.30	71	12.01			1163.38	1819.85
		SM ^11^	83	95			73.79	71	10.72			877.78	1526.77
		SL	65	125			46.34	52	27.98			1834.38	2406.92
**Varus**		CT	80	110			63.04	52	24.34			1186.83	1772.53
		SM	95	95			69.98	52	21.99			923.15	1517.88
		SL	35	125			29.35	78	9.73			2895.92	3563.87
**Valgus**		CT	50	110			48.91	78	8.31			1529.42	2199.11
		SM	65	95			58.70	78	7.42			1100.58	1771.38
^1^ Center of Rotation; ^2^ Horizontal distance between COR and vector of gluteus medius; ^3^ Horizontal distance between COR and body weight vector; ^4^ Perpendicular distance between COR and vector of gluteus medius; ^5^ Gluteus medius vector angle with horizontal axis; ^6^ Total force vector angle with vertical axis; ^7^ Gluteus medius vector; ^8^ Total force vector; ^9^ Super Lateral COR location; ^10^ Centered COR location; ^11^ Super Medial COR location.													

**Table 2:** Mesh liner details for FEA in all geometries.

Liner Thickness (mm)	Femoral Head (mm)	Element Type/Mesh Quality	Elements Size (mm)	Total Elements	Total Nodes	Element Acept. Ratio < 3 (%)
5.3	32	Solid Tetrahedron/High quality	1.14319	55,010	82,156	99.1
7.3				77,960	113,960	99.1
11.3				137,728	196,708	99.5
5.3	36			66,777	99,442	99.1
7.3				95,977	139,850	99.3
11.3				159,946	228,141	99.5

**Table 3:** (**a**) Statistical summary for VM Stress variable dependent. (**b**) ANOVA Analyses for VM Stress dependent variable (MPa). (**c**) Statistical coefficients for VM Stress dependent variable.

**(a)**						
	**R**	**R Square**	**Adjusted R Square**	**Std. Error of the Estimate**		
	0.755	0.570	0.558	4.409221380		
**(b)**						
	**Sum of Squares**	**df**	**Mean Square**		**F**	***p*-value**
Regression	5394.873	6	899.146		46.249	<0.0001
Residual	4063.218	209	19.441		-	-
Total	9458.091	215	-		-	-
**(c)**						
	**B**	**Std. Error**	**Beta**		**t**	***p*-value**
(Constant)	23.240	5.417			4.290	<0.0001
Acetabular Fixation	8.303	0.600	0.627		13.837	<0.0001
Cervical-Diaphyseal Angle	1.711	0.367	0.211		4.656	<0.0001
Thickness Liner	−2.321	0.367	−0.286		−6.317	<0.0001
COR	−0.986	0.367	−0.122		−2.684	0.008
Head Material	−1.521	0.600	−0.115		−2.536	0.012
Head Diameter	−0.493	0.150	−0.149		−3.287	0.001

**Table 4:** Comparative of statistical influence (*p*-value) among several authors. COR (L) values for Lateral positioning; COR (S) values for Superior positioning.

Author	COR (L)	COR (S)	Thickness	Head Size	Head Material
Gerogiades, 2010	0.001	0.049	-	-	-
Hirakawa, 2001	<0.0001	0.39	-	-	-
Sato, 2012	-	-	-	-	0.45
Garvin, 2015	-	-	-	-	0.58
Gwynne-Jones, 2009	-	-	-	0.21	0.6
Bragdon, 2012	-	-	-	0.23–0.90	-
Lachiewicz, 2016	-	-	-	0.593	-
Teeter, 2018	-	-	-	<0.001/0.055	0.316
Astion, 1996	-	-	0.03	-	-
Shen, 2011	-	-	0.17	0.19–0.64	-
Current study			<0.0001	0.001	0.012

## Data Availability

The data used to support the findings of the present study are available from the corresponding author upon request.
